# Recurrent Hypoglycemia Impaired Vascular Function in Advanced T2DM Rats by Inducing Pyroptosis

**DOI:** 10.1155/2022/7812407

**Published:** 2022-07-23

**Authors:** Minghao Luo, Yu Hu, Dingyi Lv, Lingyun Xie, Shenglan Yang, Deyu Zuo, Yuzhou Xue, An He

**Affiliations:** ^1^Division of Cardiology, The First Affiliated Hospital of Chongqing Medical University, Chongqing, China; ^2^Department of Rehabilitation Medicine, Chongqing Traditional Chinese Medicine Hospital, Chongqing, China

## Abstract

**Background:**

Hypoglycemia is a dangerous side effect of intensive glucose control in diabetes. Even though it leads to adverse cardiovascular events, the effects of hypoglycemia on vascular biology in diabetes have not been adequately studied.

**Methods:**

Aged Sprague-Dawley rats were fed a high-fat diet and given streptozotocin to induce type 2 diabetes mellitus (T2DM). Acute and recurrent hypoglycemia were then induced by glucose *via* insulin administration. Vascular function, oxidative stress, and pyroptosis levels in aortic tissue were assessed by physiological and biochemical methods.

**Results:**

Hypoglycemia was associated with a marked decrease in vascular function, elevated oxidative stress, and elevated pyroptosis levels in the thoracic aorta. The changes in oxidative stress and pyroptosis were greater in rats with recurrent hypoglycemia than in those with acute hypoglycemia.

**Conclusions:**

Hypoglycemia impaired vascular function in aged rats with T2DM by inducing pyroptosis. The extent of injury increased with the duration of blood glucose fluctuation.

## 1. Introduction

Type 2 diabetes mellitus (T2DM) is characterized by hyperinsulinemia and hyperglycemia and is the most common type of diabetes mellitus, and the vascular inflammation that accompanies T2DM usually leads to a variety of serious complications [1, 2]. The prevalence of T2DM increases with age, and current studies show that the elderly have a relatively high incidence and an increased risk of serious complications [1–6]. Glucose control therapy is the preferred clinical intervention for T2DM. Intensive glucose control can result in hypoglycemia, a dangerous side effect that can lead to adverse cardiovascular events, and elderly patients are at increased risk [4, 6]. The pathophysiology of hypoglycemia includes increased oxidative stress that can result in vascular injury, and recurrent hypoglycemia can interfere with vascular homeostasis, especially endothelial function [4]. Although an association between hypoglycemia and vascular dysfunction in diabetes has been shown, the underlying molecular mechanisms remain elusive and controversial. Endothelium-dependent vasodilatation is regulated by endothelial nitric oxide synthase (eNOS). As hypoglycemia in diabetes interferes with endothelial function, including endothelium-dependent vasodilation, eNOS activity may be affected [7–14].

Pyroptosis is a type of inflammatory programmed cell death. It is mediated by gasdermin (GSDM) and is characterized by inflammasome activation, caspase activation, and formation of cell membrane pores [15–17]. GSDMD-mediated pyroptosis has a role in the occurrence and development of diabetic cardiomyopathy and diabetic nephropathy [15, 18, 19]. Although the relationship between diabetic vascular injury and pyroptosis is not well established, current studies prompted us to clarify the significance of pyroptosis in diabetic vascular injury, the initial engine in pathological progress of diabetes.

Diabetic hypoglycemia promotes mitochondrial dysfunction in vascular cells that consequently increases the levels of reactive oxygen species (ROS) [20–23]. Increased expression of proinflammatory cytokines stimulates inducible nitric oxide synthase (iNOS) expression, which results in the aggravation of oxidative stress and worsening of chronic inflammation and endothelial dysfunction in diabetes [24, 25]. Oxidative stress-mediated activation of nucleotide-binding domain and leucine-rich repeat-containing receptor 3 (NLRP3) inflammasomes occurs in pyroptosis [26–28]. Hence, we hypothesized that hypoglycemia may induce pyroptosis by increasing oxidative stress that results in vascular dysfunction.

## 2. Material and Methods

### 2.1. Animals

Male 12-month-old Sprague-Dawley rats weighing 380–420 g were purchased from the Experimental Animal Center of Chongqing Medical University. All animal procedures were carried out following the guidelines on the China Animal Protection Law and were approved by the Institutional Ethics Committee of Chongqing Medical University. Animals were kept in a 12 h light–dark cycle at 22–25°C with *ad libitum* access to food and water. The aged rats were randomly divided into four groups (*n* = 6): an aged control, T2DM model (DM), T2DM hypoglycemia (H-DM), and T2DM recurrent hypoglycemia (RH-DM). Rats were fed a high-fat diet (HFD) combined with streptozotocin (STZ) to induce T2DM. Rats in the diabetes groups were fed an HFD (Nantong, Jiangsu, China) for 12 weeks followed by intraperitoneal injection of 35 mg/kg STZ (Sigma, MO, USA) in pH 4.2–4.5 citric acid [29, 30]. The citrate buffer vehicle was injected into control rats. Twelve weeks after STZ injection, rats with three consecutive random blood glucose levels > 16.7 mmol/L and diabetes behavior changes (e.g. increased food intake, increased urination, and weight loss) were considered successful T2DM models.

### 2.2. Diabetic Rat Hypoglycemia Model

**Figure 1:**
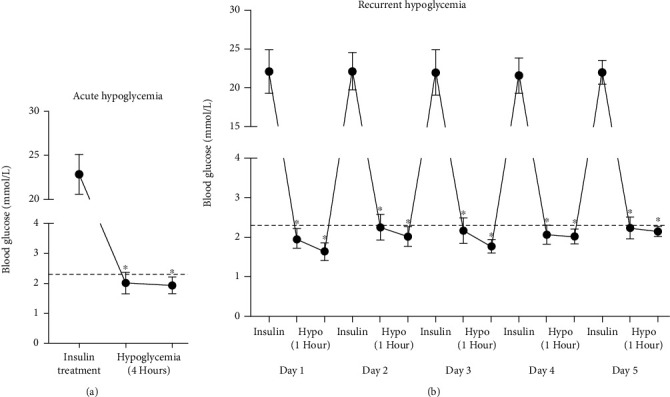
Glucose levels in T2DM model rats and insulin-induced acute or recurrent hypoglycemia; ^∗^*p* < 0.05 pretreatment vs. posttreatment.

To induce severe hypoglycemia, diabetic rats were injected with insulin (Wanbang, China) 10.0 units/kg at 8:00–9:00 in the morning after an overnight fast. Control rats were given an equal volume of phosphate buffered saline (PBS) instead of insulin. The diabetic rat hypoglycemia model (Figure 1) included an acute hypoglycemia group (hypoglycemia for 4 h/1 day) and a recurrent hypoglycemia group (hypoglycemia for 1 h/5 days). After insulin injection, blood samples were obtained by tail prick for blood glucose monitoring every 30 min to ensure that the rats maintained the glucose levels in severe hypoglycemia (<2.3 mmol/L). Rats were given 50% glucose in PBS to terminate the hypoglycemia episode. None of rats experienced seizures or coma during hypoglycemia attack. All rats were sacrificed by sodium pentobarbital anesthesia, and blood samples and aortas were collected for subsequent experiments.

### 2.3. Western Blot Assays and Antibodies

Thoracic aortas were isolated, immersed in liquid nitrogen, and then immediately transferred to a −80°C refrigerator until used in western blot assays. Proteins were separated by 10% sodium dodecyl sulfate polyacrylamide gel electrophoresis and incubated with primary antibodies: eNOS (1 : 1000, ab300071, Abcam), iNOS (1 : 1000, ab283655, Abcam), NOX2 (1 : 2000, 19013-1-AP, ProteinTech), NOX4 (1 : 2000, 14347-1-AP, ProteinTech), p-p65 (1 : 1000, 3033, Cell Signaling Technology), p65 (1 : 1000, 8242, Cell Signaling Technology), NLRP3 (1 : 1000, NBP2-12446, NOVUS), ASC (1 : 1000, sc-514414, Santa Cruz), Caspase-1 (1 : 1000, bs-10743R, Bioss), cGAS (1 : 1000, NBP3-16666, NOVUS), STING (1 : 1000, CST50494, Cell Signaling Technology), GSDMD (1 : 1000, NBP2-33422, NOVUS), Bax (1 : 1000, 50599-2-Ig, ProteinTech), Bcl-2 (1 : 1000, 26593-1-AP, ProteinTech), and *β*-actin (1 : 5000, 20536-1-AP, ProteinTech). The membranes were then incubated with horseradish peroxidase-conjugated anti-rabbit or anti-mouse IgG (1 : 5000, ProteinTech) secondary antibodies for 2 h at 37°C and visualized a with chemiluminescent reagent kit (Beyotime, Shanghai, China). The lane densities were read with a Bio-Rad imaging system.

### 2.4. Oxidative Stress Determination

Blood samples were collected with vacuum hemostix, and the sera were stored at −80°C. Antioxidant and oxidative stress indicators in serum were assayed with reagent kits provided by the Nanjing Jiancheng Bioengineering Institute (Nanjing, China) [31]. Superoxide dismutase (SOD) activity was assayed by the hydroxylamine method. Glutathione peroxidase (GSH-Px) activity was assayed by a colorimetric method. Malondialdehyde (MDA) was assayed by the thiobarbituric acid method.

### 2.5. Vascular Reactivity Tests

Thoracic aortas were removed and placed in an oxygen-containing physiological salt solution (PSS) buffer [32]. Adherent fat and connective tissues were removed, and the vascular tissue was cut into 3 mm rings to measure vascular tone as previously described. Briefly, the aortic rings were mounted horizontally on an isometric force transducer (DMT620 Multi Wire Myograph; DMT) in chambers filled with 5 mL PSS buffer at 37°C and aerated with 95% O_2_ and 5% CO_2_. The aortic rings were allowed to equilibrate for 90 min at an initial tension of 1.5 g. Concentrations of 10^−9^ to 10^−5^ M acetylcholine (ACh) for endothelium-dependent relaxation, sodium nitroprusside (SNP) for endothelium-independent relaxation, and phenylephrine (PE) were added and concentration–response curves were plotted. Vascular function was reported as the EC_50_, the concentration that produced 50% of the maximum response, *E*_max_, the maximum response, and area under the curve (AUC), which are determined by nonlinear regression analysis using GraphPad version 9.0.

### 2.6. Vascular Morphology

A section of the aorta was dissected from each rat, fixed in 4% paraformaldehyde, embedded in paraffin, sectioned at 5 *μ*m, and mounted on slides. The slides were transferred to an oven and baked for 30 min at 60°C to melt the wax, which was then removed by treatment with xylene for 20 min. Tissue sections were rehydrated in a descending ethanol series of 100% for 5 min and 95%, 90%, 80%, and 70% for 3 min each. After washing three times in PBS, the sections were stained with hematoxylin and eosin or incubated with a primary antibody. All images were captured with a Leica DM4B upright microscope (Leica Inc., Germany).

### 2.7. Immunohistochemical Staining

After dewaxing and rehydration, tissue sections were placed in citrate buffer and boiled for 5 min for antigen retrieval, washed twice with PBS, and blocked with 5% goat serum for 30 min. Tissue sections were incubated overnight with primary antibodies overnight at 4°C, washed three with PBS, incubated for 1 h at 37°C with secondary antibodies, and then washed three times with PBS. After diaminobenzidine color development for 5 min, the slides were washed with distilled water to remove float color, and the tissue was counterstained with hematoxylin for 10 s. The tissue was dehydrated in an ethanol gradient with 3 min at each concentration, cleared in xylene for 5 min, mounted with resin, and observed by light microscopy (Leica Inc., Germany). The primary antibodies used were eNOS (1 : 200, ab300071, Abcam), iNOS (1 : 200, ab283655, Abcam), NLRP3 (1 : 200, NBP2-12446, NOVUS), and GSDMD (1 : 200, NBP2-33422, NOVUS). Area quantitative assessment of immunohistochemical staining was performed by the ImageJ 1.8 software (USA).

### 2.8. Immunofluorescence Staining

Aorta sections were permeabilized with 0.1% Triton X-100 in PBS for 20 min, blocked with 5% goat serum for 1 h, and incubated with antibodies overnight at 4°C. The tissue sections were then incubated for 5 minutes with 4′,6-diamidino-2-phenylindole (DAPI) and then for 1 h with fluorescence-conjugated secondary antibodies (Beyotime, Shanghai, China) in the dark at 37°C. Tissues were observed by fluorescence microscopy (Leica Microsystems, Germany). The antibodies used were 8-OHdG (1 : 200, bs-1278R, Bioss), NLRP3 (1 : 200, NBP2-12446, NOVUS), and GSDMD (1 : 200, NBP2-33422, NOVUS). Fluorescence intensity was quantified using the ImageJ 1.8 software.

### 2.9. Data Analysis

Numeric values were reported as the means ± standard deviation. Normality of the distribution of data was assessed by the Shapiro-Wilk normality test. To calculate the comparisons between 2 groups, normally or nonnormally distributed data were compared using the unpaired 2-tailed Student *t* tests or the Mann–Whitney *U* test, respectively. To calculate the comparisons between multiple groups (≥3 groups), normally or nonnormally distributed data were compared using one-way analysis of variance (ANOVA) followed by the Bonferroni post hoc test or Kruskal-Wallis test followed by the Dunn post hoc test, respectively. The statistical analysis was performed with GraphPad Prism 9.0. *p* values < 0.05 were considered statistically significant.

## 3. Results

### 3.1. Establishment of Acute or Recurrent Hypoglycemia Models in Aged T2DM Rats

The primary purpose of the study was to establish a reliable rat model of T2DM hypoglycemia. As shown in Figure 1, diabetes was induced by STZ (10.0 units/kg), which destroyed pancreatic *β*-cells and induced insulin resistance when combined with an HFD. We observed a significant increase in blood glucose levels (22.8 ± 2.2 mM). As shown in Figure 1(a), insulin treatment resulted in severe hypoglycemia (2.0 ± 0.3 mM/L) in the H-DM group that was maintained for 4 h. As shown in Figure 1(b), the blood glucose values in the RH-DM group reached the target levels for 5 consecutive days. Subsequent procedures were based on this model.

### 3.2. Vascular Function, eNOS, and iNOS Expression in Response to Acute and Recurrent Hypoglycemia in Aged T2DM Rats

**Figure 2:**
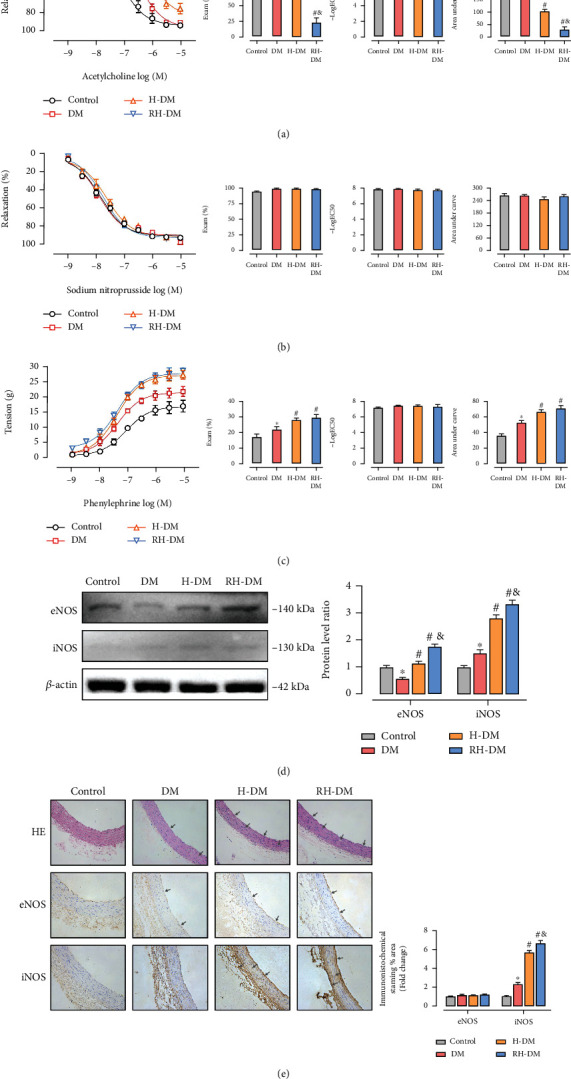
Vascular function, eNOS, and iNOS expression in aortas from aged T2DM rats in response to acute and recurrent hypoglycemia. (a) ACh- and (b) SNP-induced relaxation responsiveness and (c) PE-induced contraction responsiveness in controls, aged diabetes (DM), acute hypoglycemia (H-DM), and recurrent hypoglycemia (RH-DM) groups were determined by testing the reactivity of aorta rings. Vascular function was reported by EC_50_, *E*_max_, and the AUC. eNOS and iNOS protein expressions were assayed in (d) western blots and (e) by immunohistochemical staining. Aorta morphology was evaluated by hematoxylin and eosin staining; ^∗^*p* < 0.05 DM vs. control; ^#^*p* < 0.05 H-DM, RH-DM vs. DM; ^&^*p* < 0.05 H-DM vs. RH-DM.

We first evaluated vascular function in the study groups, which was reported as EC_50_ and *E*_max_. The effect of hypoglycemia on vasodilation of the aorta was evaluated by ACh- and SNP-induced relaxation responsiveness. As shown in Figures 2(a) and 2(b), after the tension of PE-mediated vasoconstriction was balanced, ACh or SNP (10^−9^–10^−5^ M) was added in half-log increments. Compared with control, ACh-induced dilation function was impaired in DM (−log EC_50_: control 7.10, DM 6.44; AUC: control 199.0, DM 155.0; *p* < 0.05). Acute and recurrent hypoglycemia significantly exacerbated ACh-induced vasodilation of the aorta, compared with DM (−log EC_50_: H-DM 6.10, RH-DM 5.88; AUC: H-DM 102.9, RH-DM 30.3; *p* < 0.05). ACh-induced relaxation in the group with recurrent hypoglycemia was worse than that in the group with acute hypoglycemia (*p* < 0.05). There were no significant differences in the SNP-induced vasodilation function among the groups (Figure 2(b); *p* > 0.05). The results indicate that hypoglycemia in the model rats impaired endothelium-dependent relaxation and that recurrent hypoglycemia had a potentially more harmful effect on the endothelium-dependent relaxation.

Studies have shown an enhanced response to catecholamines in DM [33–35]. We therefore explored the role of hypoglycemia on the contraction of the aortas of the model rats (Figure 2(c)). The *E*_max_ of PE-induced contraction was increased in DM rats compared with controls (*E*_max_: control 16.96, DM 22.06; AUC: control 35.37, DM 52.12; *p* < 0.05), but the EC_50_ was not significantly affected (*p* < 0.05). The results showed that both acute and recurrent hypoglycemia significantly raised PE-induced contraction of aortas from model rats (*E*_max_: H-DM 27.74, RH-DM 29.23; AUC: H-DM 66.19, RH-DM 70.82; *p* < 0.05).

We also determined the expression of two nitric oxide synthases, eNOS, and iNOS, in the aorta. As shown in Figure 2(d), eNOS protein expression decreased, and that of iNOS increased in DM compared with control (*p* < 0.05). Interestingly, insulin treatment significantly increased eNOS expression in the hypoglycemia groups compared with DM (*p* < 0.05), and expression was higher in the RH-DM than in the H-DM group (DM 0.54-, H-DM 1.13-, and RH-DM 1.76-fold compared with controls; *p* < 0.05). H-DM and RH-DM significantly increased iNOS expression in the aortas (DM 1.51-, H-DM 2.80-, and RH-DM 3.34-fold compared with controls; *p* < 0.05).

Vascular morphology analysis revealed that inflammatory cell infiltration of the intima and subintimal layers was significantly increased in diabetic compared with nondiabetic rats, with disruption of the morphology of the endothelium and smooth muscle layers (gray arrows). Importantly, inflammatory cell infiltration and disruption of endothelial structures were worse in the H-DM and RH-DM groups (Figure 2(e)). The results of eNOS and iNOS immunohistochemical staining are shown in Figure 2(e). Acute and recurrent hypoglycemia significantly increased the area of iNOS in DM, especially in the endothelium and perivascular tissue. Rupture of the endothelial layers was obvious in the hypoglycemic groups (gray arrows).

### 3.3. Oxidative Stress in Aged T2DM Rats with Acute and Recurrent Hypoglycemia

**Figure 3:**
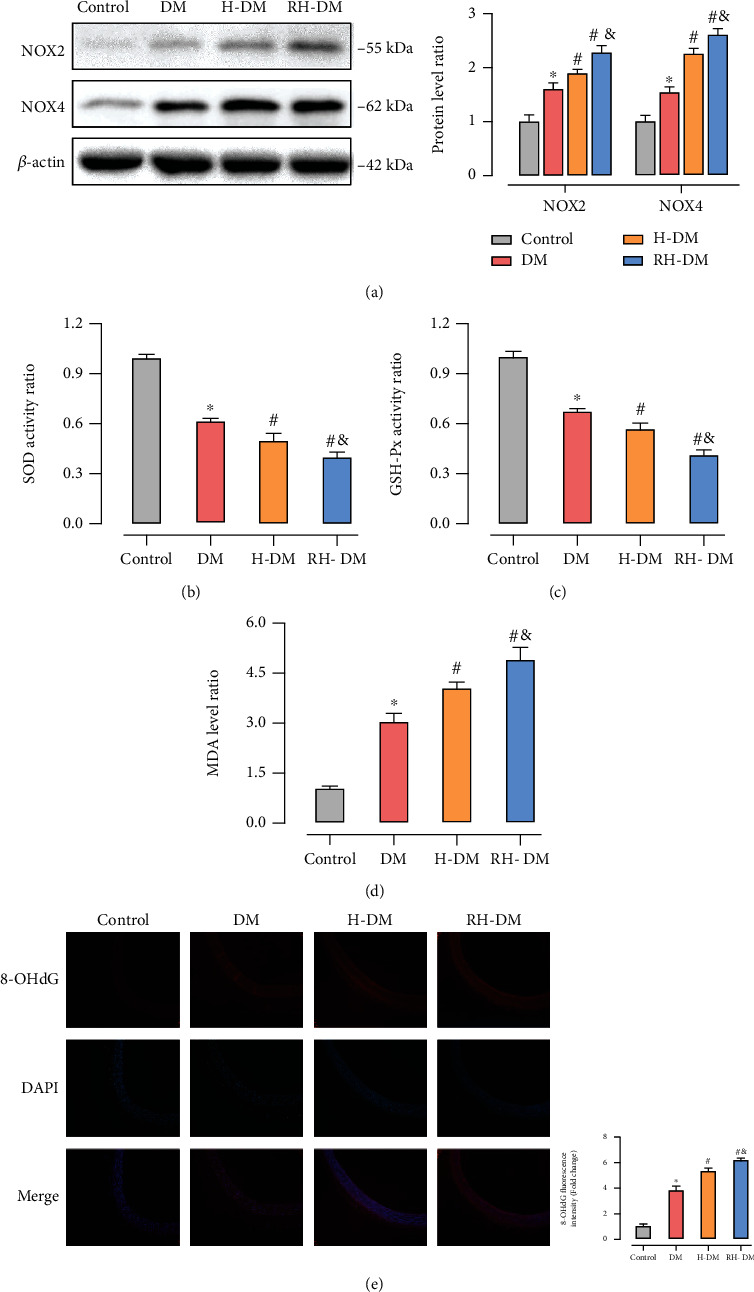
Oxidative stress in the aorta in response to acute and recurrent hypoglycemia in aged T2DM rats. NOX2/4 expression was assayed by (a) western blotting, (b) serum levels of SOD, (c) GSH-Px, and (d) MDA were tested. (e) 8-OHdG location and expression were determined by immunofluorescence; ^∗^*p* < 0.05 DM vs. control; ^#^*p* < 0.05 H-DM, RH-DM vs. DM; ^&^*p* < 0.05 H-DM vs. RH-DM.

NADPH oxidase (NOX) 2 and 4 are key enzymes involved in electron transfer in the cell membrane and are sources of ROS in the cardiovascular system [36, 37]. To determine the level of oxidative stress, we assayed the expression of NOX2 and NOX4 in the aorta by western blotting. As shown in Figure 3(a), H-DM and RH-DM significantly increased NOX2 and NOX4 expressions in the aortas of model rats (NOX2: DM 1.59-, H-DM 1.89-, and RH-DM 2.20-fold compared with controls; NOX4: DM 1.55-, H-DM 2.22-, and RH-DM 2.50-fold compared with control). To further investigate the effects of hypoglycemia on oxidative stress, SOD and GSH-Px (antioxidant enzymes active against free radicals), and MDA (an indicator of lipid peroxidation) were assayed in serum (Figures 3(b)–3(d)). Antioxidant activity was significantly higher in the RH-DM group than in the other groups (*p* < 0.05). To better understand the effects of diabetic hypoglycemia on vascular damage and the underlying mechanisms, we assayed 7,8-dihydro-8-oxo-2-deoxyguanosine (8-OHdG) in aorta tissue by immunofluorescence. 8-OHdG is formed in aerobic organisms by the oxidation of DNA and reflects the level of cellular oxidative stress and the degree of mitochondrial damage [38, 39]. 8-OHdG levels were higher in the hypoglycemia groups than in the DM group, and all tissues of the aorta were involved (Figure 3(e)).

### 3.4. The Nuclear Factor Kappa B (NF-*κ*B), NLRP3, and Cyclic Guanosine Monophosphate–Adenosine Monophosphate Synthase– (cGAS–) Stimulator of Interferon Genes (STING) Pathways in Acute and Recurrent Hypoglycemia in Aged T2DM Rats

**Figure 4:**
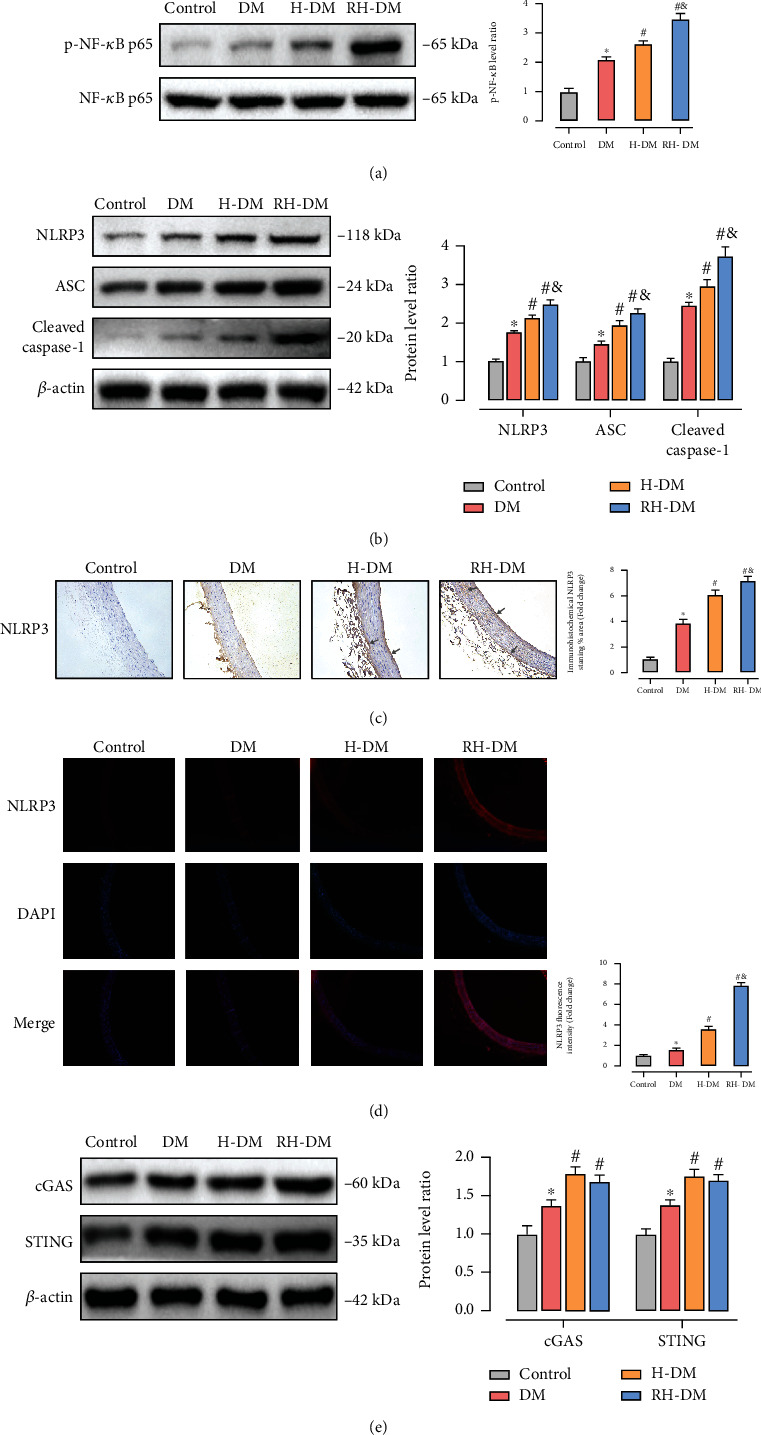
NF-*κ*B, NLRP3, and cGAS–STING pathway activity of the aortas in response to acute and recurrent hypoglycemia in aged T2DM rats. (a) p-p65, NLRP3, ASC, (b) cleaved caspase-1, cGAS, and (e) STING expression were assayed by western blotting. The expression and location of NLRP3 were determined by (c) immunohistochemistry and (d) immunofluorescence; ^∗^*p* < 0.05 DM vs. control; ^#^*p* < 0.05 H-DM, RH-DM vs. DM; ^&^*p* < 0.05 H-DM vs. RH-DM.

The NF-*κ*B inflammatory pathway promotes NLRP3 transcription, which in turn causes vascular dysfunction in diabetes [40, 41]. As shown in Figure 4(a), the percentage of phosphorylated NF-*κ*B core protein p65 increased to 2.57-fold in the aortas from rats with acute hypoglycemia and to 3.48-fold in those with recurrent hypoglycemia compared with control rats at baseline. The role of pyroptosis induced by NLRP3 inflammasomes in diabetic vascular dysfunction has been confirmed. We used western blotting to assay the expression of NLRP3, apoptosis-associated speck-like protein (ASC), and cleaved caspase-1 in aorta tissue (Figure 4(b)). The related proteins detected in the H-DM and RH-DM groups were activated compared with the DM group (*p* < 0.05). NLRP3 pathway activity was higher in the RH-DM group than in the other groups (NLRP3: DM 1.74-, H-DM 2.09-, and RH-DM were 2.45-fold compared with control; *p* < 0.05). The expression and location of NLRP3 were determined by immunohistochemistry (Figure 4(c)) and immunofluorescence (Figure 4(d)). NLRP3 expression was stronger in rats with hypoglycemia compared with the DM group, and all the vessel layers were involved (gray arrows).

cGAS–STING is a DNA sensor that triggers the innate immune response. cGAS produces the second messenger cGAMP and activates STING, which participates in autoimmune and inflammatory diseases [42]. The pathway was activated in the aortas of elderly diabetic rats (*p* < 0.05), and the difference between recurrent and acute hypoglycemia was not significant (cGAS: DM 1.36-, H-DM 1.78-, and RH-DM 1.62-fold compared with control).

### 3.5. Pyroptosis in Acute and Recurrent Hypoglycemia

**Figure 5:**
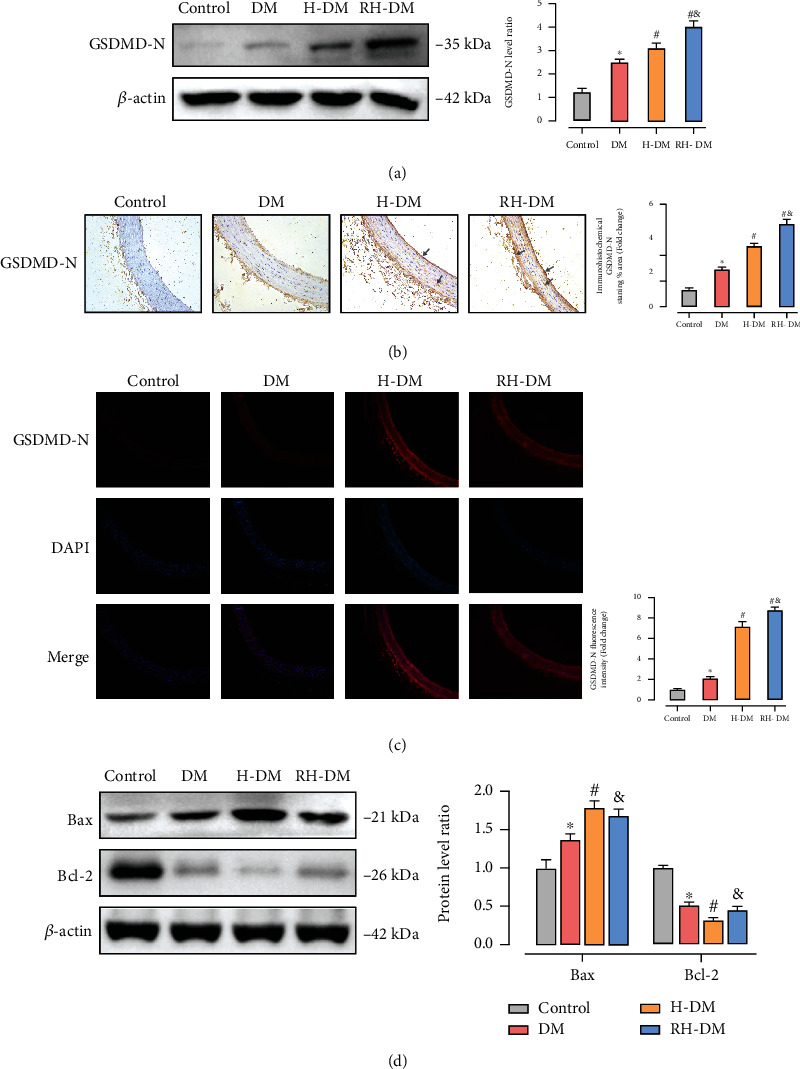
Pyroptosis in the aortas in response to acute and recurrent hypoglycemia in aged T2DM rats. (a) GSDMD-N, Bax, and (b) Bcl-2 expression were assayed by western blotting. Expression and location of GSDMD-N were identified by (b) immunohistochemistry and (c) immunofluorescence; ^∗^*p* < 0.05 DM vs. control; ^#^*p* < 0.05 H-DM, RH-DM vs. DM; ^&^*p* < 0.05 H-DM vs. RH-DM.

To confirm whether acute and recurrent hypoglycemia affected pyroptosis activity in the aortas of diabetic rats, expression of the pyroptosis-associated protein GSDMD-N was assayed by western blotting, immunohistochemistry, and immunofluorescence. As shown in Figure 5(a), GSDMD-N expression increased in the DM group compared with control (*p* < 0.05). Insulin treatment significantly increased pyroptosis in the hypoglycemia groups compared with the DM group (*p* < 0.05), and pyroptosis activity was higher with RH-DM than with H-DM (DM 2.54-, H-DM 3.08-, and RH-DM 4.04-fold compared with control). As shown in Figures 5(b) and 5(c), GSDMD-N expression was stronger in rats with hypoglycemia compared with the DM group, and all the vessel layers were involved, especially the endovascular cortex (gray arrows). To assess the influence of acute and recurrent hypoglycemia on apoptosis, the expression of proapoptotic proteins (Bax) and antiapoptotic proteins (Bcl-2) was assayed. As shown in Figure 5(d), apoptosis was increased in in aorta tissue from the H-DM compared with the DM group (*p* < 0.05). Apoptosis rates in the RH-DM and DM groups were not significantly different, but apoptosis was significantly lower in the RH-DM than in the H-DM group (Bax: DM 1.55-, H-DM 1.83-, and RH-DM 1.62-fold compared with control; Bcl-2: DM 0.50-, H-DM 0.30-, and RH-DM 0.44-fold compared with control). Acute and recurrent hypoglycemia increased GSDMD-N expression and promoted pyroptosis. Apoptosis was not increased in rats with RH-DM.

## 4. Discussion

Our study found that hypoglycemia-induced vascular dysfunction in aged diabetes was associated with an increase of pyroptosis and is the first to report the effect of hypoglycemia on pyroptosis. Hypoglycemia is a common clinical event in diabetes patients, especially those with intensive glycemic control and using insulin or other hypoglycemic agents [4, 7, 43]. Most studies of diabetic hypoglycemia have focused on cognitive dysfunction [23, 43–45]. As glucose is the primary the energy source of the adult human brain, severe hypoglycemia, a blood glucose of <2.3 mM, can lead to coma, permanent brain damage, or death. Unfortunately, most instances of clinical hypoglycemia are not occasional but are recurrent and persistent. It is urgent to reveal the underlying mechanisms of cognitive deficits that result from RH and identify new intervention targets.

Most studies have shown that hypoglycemia promotes oxidative stress-induced brain damage in diabetes patients [21, 23, 43]. We believe that such events are mainly related to vascular damage. This study focused on vascular dysfunction in DM, which is a frequent complication that can result in generalized inflammation of small vessels and even multiorgan failure. We used thoracic aortas as a representative vessel for the investigation of vascular function. The next step of our research team is to compare whether cerebrovascular and conduction vessels respond differently to hypoglycemic events.

Endothelial cells regulate vascular tension by the release of potent vasoactive substances such as nitric oxide. One causes of endothelial dysfunction is a decline in NO production [12, 32, 46]. In addition to the physiologic and biochemical findings, our study revealed impaired endothelial-dependent vasodilation responses to acute and recurrent hypoglycemia in aged diabetic rats. ACh promotes the release of factors such as NO and prostacyclin that cause relaxation. SNP directly affects vascular smooth muscle by increasing guanylate cyclase activity, resulting in an endothelium-independent relaxation [31]. We found that the response of the vascular smooth muscle to NO was not changed in the aortas of diabetes model rats and that hypoglycemia interfered with endothelium-dependent relaxation that was induced by Ach (Supplementary file 1).

It is noteworthy that eNOS and iNOS expressions were both the highest in the RH-DM group. The decoupling of eNOS induced by acute fluctuations of blood glucose in the endothelial cells of aged diabetic rats reduced the effectiveness of eNOS and resulted in an excess of peroxide anions [8–11]. Increased production of iNOS and decoupled eNOS led to accumulation of highly cytotoxic reactive nitrogen species, i.e., nitric oxide-derived compounds such as ONOO^–^, which induces vascular hyporeactivity and cell death [47]. Most previous studies focused on how hyperglycemia induced vascular events by impairing eNOS activity or inducing eNOS uncoupling in diabetes [25, 46]. They did not adequately investigate the molecular mechanisms underlying the relationship between vessel damage and hypoglycemia. This is the first report that shows the recurrent hypoglycemia-induced eNOS decoupling, and we will investigate that relationship in future studies.

The endothelium of intact aortas from RH-DM and H-DM rats was more responsive to the contractile effects of norepinephrine than aortas from controls. The mechanisms are not completely understood, but some reasons for the increased vascular responsiveness to contractile agents are deficient endothelial activity, enhanced sensitivity of calcium channels, increased sensitivity to adrenergic agonists, enhanced oxidative stress, and decreased antioxidant defenses [34]. Two major conclusions from the study results are that the relaxation response to ACh was decreased by hypoglycemia and that the contractile response of the endothelium of intact aortas from diabetic rats to catecholamine was increased.

The prevalence of diabetes increases with age and is estimated as 20% in those 65–79 years of age [1]. Most previous studies have used young animals to model diabetes. In this study, an aged T2DM model was induced by STZ combined with 12 weeks of an HFD. Age is a key factor in determining both the risk of diabetes and subsequent outcomes [48]. Changes in molecular and cellular aging processes are currently believed to be the basis of cardiovascular disease, including autophagy, inflammation, oxidative stress, DNA damage, protease inactivation, and epigenetic disorders [49]. Aging is the greatest risk factor for most chronic diseases that have increased risks of morbidity and mortality. Aging is associated with progressive impairment of metabolic pathways that affect body composition, insulin resistance, mitochondrial dysfunction, and inflammation [5]. Aging research has focused on understanding the molecular mechanisms that regulate the aging process and identifying biomarkers that could help to predict age-related changes. The rats used in this study were 18 months old when they were sacrificed, which is an age equivalent to 50–60-year-old humans, in whom the incidence of diabetes is relatively high.

The activation pyroptosis involves the recognition of NLRP3 inflammasome agonists and inflammasome assembly and activation. NLRP3 has been reported to be activated by a variety of unrelated pathogen-associated molecular patterns (PAMPs) and damage-associated molecular patterns (DAMPs), but there is no evidence that NLRP3 binds directly to those effectors. Multiple molecular and cellular events, including ion flux, mitochondrial dysfunction, release of ROS, and mitochondrial DNA (mtDNA) that induce NLRP3 stimuli, have been proposed as upstream signals for the assembly and activation of inflammasomes and caspase activation [28]. Activated caspases cleave gasdermin D at the interdomain loop to release the N-terminal pore-forming domain, which is then translocated to the plasma membrane to form pores induce pyroptosis. In contrast to other forms of cell death, pyroptosis has unique morphological and physical characteristics, including intact nuclei, cellular swelling, and plasma membrane rupture. In this study, changes in the levels of NOX2, NOX4, and 8-OHdG indicate that the release of ROS and mtDNA in model mice with hypoglycemia promoted pyroptosis (Supplementary file 3-5). Available evidence shows that activation of fatty acid oxidation followed by mtROS production may be a cause of endothelial dysfunction during hypoglycemia. cGAS is a cytoplasmic DNA biosensor that recognizes DNA from pathogens or damage. Mitochondrial metabolic stress has been reported to contribute to diabetic cardiomyopathy by mtDNA-mediated activation of the cGAS–STING pathway. Interestingly, we observed that the cGAS–STING pathway was not significantly elevated in recurrent hypoglycemia compared with acute hypoglycemia, unlike NLRP3-mediated pyroptosis. That observation requires subsequent analysis and study.

Cell death is a fundamental physiological process in organ homeostasis, coordination of immune responses, and autoimmunity. Our understanding of the mechanisms orchestrating cellular death has increased substantially, and the modalities of programmed cell death that have been described highlight the complex mechanisms that tip the balance between different cell fates. The three most well understood modalities of cell death are apoptosis, necroptosis, and pyroptosis. Cell death is a very intricate game where distinct central players have the power to tip a fragile balance from life to death and include pro- and anti-inflammatory signals in the cell environment [50]. It has been suggested that pyroptosis is a form of apoptosis, and there is evidence that the two different modes of cell death act independently. New evidence suggests that members of the gasdermin superfamily promote apoptosis by permeabilizing mitochondria and participate in the final stages of the apoptotic program by inducing secondary pyroptosis [51]. The results shown in Figure 5 indicate that acute and recurrent hypoglycemia increased GSDMD-N expression and promoted pyroptosis and that apoptosis was lower in rats with RH-DM than it was in those with H-DM. In our study, apoptosis and pyroptosis levels were different, which further demonstrates the importance of pyroptosis in hypoglycemic responses.

Current studies of the complications of hypoglycemia in diabetes have primarily focused on brain injury induced by hypoglycemia [23, 43–45]. Studies of the effects of severe hypoglycemia in humans have yielded conflicting results. Severe hypoglycemia has been shown to alter brain structure and cause significant cognitive damage in many, but not all, studies. Reasons for the discrepancy are not known, but a major contributing factor may be the extent of glycemic control (including recurrent hypoglycemia) prior to episodes of severe hypoglycemia. There is evidence that recurrent episodes of moderate hypoglycemia protect the brain against damage caused by a subsequent episode of more severe hypoglycemia, a phenomenon known as hypoglycemia preconditioning [43]. These intriguing findings suggest that recurrent bouts of moderate hypoglycemia that occur with intensive glycemic control might, paradoxically, render an individual more prone but less vulnerable to an episode of severe hypoglycemia. It is more likely that the brain adapts to low glucose, but it is by no means certain that this tendency has individual benefits. The results of our study suggest that recurrent hypoglycemia did indeed damage vascular function (Supplementary file 1-5).

The association between severe hypoglycemia and increased risk of vascular events has been acknowledged in diabetes, but the exact mechanism has rarely been explored in recent years. In summary, our study found that pyroptosis had a key role in vascular dysfunction associated with hypoglycemia induced by glycemic control in a rat diabetes model induced by STZ and an HFD.

## Data Availability

All data utilized in this study are included in this article, and all data supporting the findings of this study are available on reasonable request from the corresponding author.
